# Total weight loss induces the alteration in thyroid function after bariatric surgery

**DOI:** 10.3389/fendo.2024.1333033

**Published:** 2024-01-30

**Authors:** Ziru Tian, Yuntao Nie, Zhengqi Li, Pengpeng Wang, Nianrong Zhang, Xiaofan Hei, An Ping, Baoyin Liu, Hua Meng

**Affiliations:** ^1^ Department of General Surgery & Obesity and Metabolic Disease Center, China-Japan Friendship Hospital, Beijing, China; ^2^ School of Basic Medical Sciences, Capital Medical University, Beijing, China; ^3^ Department of Emergency, China-Japan Friendship Hospital, Beijing, China

**Keywords:** bariatric surgery, sleeve gastrectomy, Roux-en-Y gastric bypass, thyroid function, weight loss

## Abstract

**Background:**

Bariatric surgery is an effective approach to weight loss, which may also affect thyroid function. However, alteration in thyroid-stimulating hormone (ΔTSH) and thyroid hormones after bariatric surgery and the relationship between thyroid function and postoperative weight loss still remains controversial.

**Methods:**

Data were collected from euthyroid patients with obesity who underwent sleeve gastrectomy and Roux-en-Y gastric bypass from 2017 to 2022. The alterations of free thyroxine (FT4), free triiodothyronine (FT3), total thyroxine (TT4), total triiodothyronine (TT3), and TSH were calculated 1 year after surgery. Pearson correlation analysis was used to assess the correlation between the percentage of total weight loss (%TWL) and ΔTSH. Multivariable linear regression was utilized to determine the association between %TWL and ΔTSH.

**Results:**

A total of 256 patients were included in our study. The mean %TWL was 28.29% after 1 year. TSH decreased from 2.33 (1.67, 3.04) uIU/mL to 1.82 (1.21, 2.50) uIU/mL (*P* < 0.001), FT3 decreased from 3.23 ± 0.42 pg/mL to 2.89 ± 0.41 pg/mL (P < 0.001), FT4 decreased from 1.11 ± 0.25 ng/dL to 1.02 ± 0.25 ng/dL (*P* < 0.001), TT3 decreased from 1.13 (1.00, 1.25) ng/mL to 0.89 (0.78, 1.00) ng/mL (*P* < 0.001), and TT4 decreased from 8.28 ± 1.69 ug/mL to 7.82 ± 1.68 ug/mL 1 year postoperatively (*P* < 0.001). %TWL was found to be significantly correlated to ΔTSH by Pearson correlation analysis (Pearson correlation coefficient = 0.184, *P* = 0.003), indicating that the more weight loss, the more TSH declined. After adjusting for covariates in multivariable linear regression, %TWL was found to be independently associated with ΔTSH (β = 0.180 [95% confidence interval (CI), 0.048 – 0.312], *P* = 0.008). Moreover, %TWL was divided into 3 categorical groups (%TWL ≤ 25%, 25% < %TWL ≤ 35%, and %TWL > 35%) for further exploration, and was also found to be an independent predictor for ΔTSH after adjusting for covariates in multivariable linear regression (β = 0.153 [95% CI, 0.019 – 0.287], *P* = 0.025).

**Conclusion:**

TSH, FT4, FT3, TT4, and TT3 decrease significantly 1 year after bariatric surgery. The decline in TSH is independently mediated by postoperative weight loss; the more the weight loss, the more the TSH decrease.

## 1 Introduction

Obesity has emerged as a global health concern, accompanied by a variety of endocrine comorbidities, including thyroid dysfunction (1). Evidence has shown that body weight is associated with thyroid function, in which obesity can lead to a higher risk of overt or subclinical hypothyroidism (2). In contrast, weight loss may have the potential to ameliorate abnormalities in blood glucose and lipids, reduce the inflammatory state of the body, and protect the thyroid gland (3).

Bariatric surgery has been demonstrated to be an effective and permanent approach to weight loss, with sleeve gastrectomy (SG) and Roux-en-Y gastric bypass (RYGB) being the most commonly performed procedures (4, 5). Based on previous literature, bariatric surgery was associated with the relief of thyroid dysfunction, alleviating overt or subclinical hypothyroidism, and reducing the need for thyroid hormone-lowering therapy (6, 7). These effects may be attributed to the declines in serum levels of leptin, adipokines, and ghrelin levels after surgery (8, 9). However, changes in thyroid-stimulating hormone (TSH) after bariatric surgery reported by different studies varied dramatically. TSH levels were found to decline significantly following bariatric surgery in the majority of studies involving patients with subclinical or overt hypothyroidism (10–12). Similar results have been noted in a number of researches involving patients with normal thyroid function (13–15). Nonetheless, a retrospective study by MacCuish et al. (16) including 55 euthyroid patients undergoing RYGB found that TSH levels remained steady after 2 years. Dall’Asta et al. (17) conducted a retrospective study in 258 euthyroid patients who had received gastric banding also failed to find a significant change in TSH postoperatively. The effect of bariatric surgery on the serum levels of free triiodothyronine (FT3) and free thyroxine (FT4) is not yet fully understood. Patients from different countries who underwent various surgical procedures were reported to exhibit elevated, unchanged, or decreased FT3 and FT4 levels following surgery in prior studies (15, 18, 19). A recent meta-analysis in 2017 revealed that FT3 decreased in euthyroid individuals after bariatric surgery, while FT4 remained unchanged (20). Therefore, the impact of weight loss following bariatric surgery on thyroid function in euthyroid patients with obesity remains controversial.

The aim of our study was to investigate the alterations in TSH and thyroid hormones 1 year after bariatric surgery in euthyroid patients with obesity, and explore the relationship between the percentage of total weight loss (%TWL) and alterations of TSH (ΔTSH).

## 2 Materials and methods

### 2.1 Study design and participants

This is a retrospective observational analysis of prospectively collected data of patients with obesity who underwent SG or RYGB at China-Japan Friendship Hospital from September 2017 to May 2022. All the patients were selected for bariatric surgery according to the Chinese Surgical Guideline for Obesity and Type 2 Diabetes by the Chinese Society for Metabolic and Bariatric Surgery (CSMBS) (21). The inclusion criteria were (1) patients with obesity (BMI ≥ 27.5 kg/m^2^ according to Chinese guidelines (21), and guidelines from the American Society for Metabolic and Bariatric Surgery and International Federation for the Surgery of Obesity and Metabolic Disorders (5)), (2) age between 16 and 65 years, (3) complete preoperative and postoperative thyroid function tests, and (4) normal preoperative TSH (< 0.50 uIU/mL or > 4.80 uIU/mL) and FT4 (< 0.58 ng/dL or > 1.75 ng/dL) levels. Patients with a history of pituitary and/or thyroid disease, abnormal thyroid hormone levels, a history of drug application that might affect thyroid function (e.g. amiodarone, lithium, anticonvulsants, and glucocorticoids), and those who had undergone a second bariatric surgery were excluded from the study. Patients with a metformin usage history were not excluded because it is commonly used in patients with obesity and type 2 diabetes mellitus (T2DM), despite it might affect thyroid function.

This study was performed in accordance with the Helsinki Declaration and was approved by the Institutional Review Board (IRB) of China-Japan Friendship Hospital (2021-112-K70). Informed consent was waived by the IRB because the study was observational and noninvasive.

### 2.2 Variables collection

All sociodemographic variables were extracted from the electronic medical record system, including sex, age, height, weight, waist circumstance, hip circumstance, type of surgery, T2DM, hypertension, systolic blood pressure, diastolic blood pressure, smoking history, and alcohol consumption. The T2DM was diagnosed according to the American Diabetes Association guidelines (22), including fasting plasma glucose (FPG) ≥ 7.0 mmol/L and/or 2-h plasma glucose ≥ 11.1 mmol/L during oral glucose tolerance test and/or glycated hemoglobin (HbA1c) ≥ 6.5% and/or patients with classic symptoms of hyperglycemia or hyperglycemic crisis, a random plasma glucose ≥ 11.1 mmol/L and/or a diagnosis of T2DM in the past. Diagnosis of hypertension was based on systolic blood pressure ≥ 140 mmHg and/or diastolic blood pressure ≥ 90 mmHg and/or a history of hypertension (23).

The biochemical variables of blood samples were collected and examined within a week preoperatively, including TSH, thyroid hormones, lipid profiles, and glycemic profiles, et al. The serum levels of TSH, FT4, FT3, total thyroxine (TT4), and total triiodothyronine (TT3) were measured by electrochemiluminescence immunoassay. The enzymatic colorimetric method was utilized to measure the serum levels of FPG, while HbA1c was measured by high-performance liquid chromatography. Total cholesterol (TC) and total triglyceride (TG) were measured by standard enzymatic methods. The serum levels of high-density lipoprotein cholesterol (HDL-C) and low-density lipoprotein cholesterol (LDL-C) were measured by the direct method. The normal ranges for all biochemical variables are listed in **Supplementary Table 1**.

In addition, the alterations of clinical parameters were calculated by subtracting the levels at baseline from the levels at 1 year, and the %TWL was calculated from the follow-up data.

### 2.3 Surgical procedures

Symmetric three-port laparoscopic SG and RYGB were performed as described in our previous studies (24, 25). The patients were positioned supine, with their arms extended laterally and their legs joined. Laparoscopic SG was performed over a 36-Fr bougie, with the sleeve beginning 4-6 cm from the pylorus. Laparoscopic RYGB was performed using a standard 30 mL pouch, a biliopancreatic limb measuring 100 cm, and a Roux limb measuring 100 cm. Gastrojejunostomy was made with a linear stapler with hand-sewn defect closure. Patients with a long duration of T2DM, weakened islet function, or severe gastroesophageal reflux disease symptoms were recommended RYGB as a priority, but patient preference was also considered.

### 2.4 Postoperative management

All patients were instructed to take a daily oral micronutrient/multivitamin supplement (Centrum^®^), which contained the following nutrients: vitamin A (4000 IU), vitamin D (400 IU), vitamin E (30 IU), vitamin B1 (1.5 mg), vitamin B2 (1.7 mg), vitamin B6 (2 mg), vitamin C (60 mg), vitamin B12 (6 μg), folate (400 μg), calcium (162 mg), iron (18 mg), zinc (15 mg), and magnesium (100 mg). This supplementation regimen was advised to be continued for at least 1 year for patients undergoing SG, and a lifelong duration for patients undergoing RYGB.

Follow-up were scheduled at 3 months, 6 months, 1 year postoperatively, and subsequently on an annual basis. Various parameters including weight, blood pressure, and biochemical tests were regularly monitored during these follow-ups.

### 2.5 Statistical analysis

Continuous variables were presented as means ± standard deviation (SD) for normal data and medians with interquartile range for non-normal data, and the categorical variables were shown as frequency (percentage). The changes between clinical variables at baseline and 1 year postoperatively were tested by paired sample t-test or the Mann-Whitney U test. Differences between groups were evaluated by analysis of variance (ANOVA) tests for continuous data. Correlation between ΔTSH and %TWL was determined using the Pearson correlation analysis, in which a *P* < 0.05 indicated the presence of a significant correlation. The %TWL was utilized as both continuous and categorical variables (%TWL group) to explore their relationship with ΔTSH. The %TWL group was divided as follows: %TWL ≤ 25%, 25% < %TWL ≤ 35%, and %TWL > 35%. The univariable and multivariable stepwise linear regression was conducted to examine the association between ΔTSH and %TWL. Three linear regression models were performed. The Model 1 was unadjusted; Model 2 adjusted for sex, age, and body mass index (BMI); The Model 3 further adjusted for waist-to-hip ratio, type of surgery, T2DM, metformin use, hypertension, smoking history, and alcohol consumption. In addition, the subgroup analysis was conducted to assess whether the potential covariates (sex, age, BMI, T2DM, metformin use, and hypertension) modified the relationship between ΔTSH and %TWL using multivariable linear regression models with full adjustment in Model 3. The significance level was a *P* < 0.05 in all tests. Data analysis was carried out with SPSS version 24 and R 4.1.3 software (https://r-project.org/).

## 3 Results

### 3.1 Patient characteristics

The clinical characteristics of patients are summarized in **Table 1**. A total of 256 patients were included in the study, 64.8% of patients were female, and their median age was 35.00 (29.00, 43.75) years. The baseline average BMI was 38.37 ± 7.24 kg/m^2^, and the mean waist-to-hip ratio was 0.96 ± 0.08. Two hundred and two (78.9%) patients underwent SG, and 54 (21.1%) underwent RYGB. Among these patients, 148 (57.8%) were diagnosed as T2DM, and 75 (29.30%) had a history of metformin use. One-hundred and nine (46.4%) patients were diagnosed as hypertension, and the mean systolic blood pressure was 138.08 ± 19.57 mmHg, and the diastolic blood pressure was 87.16 ± 15.28 mmHg.

**Table 1:** Patient characteristics.

Variables	Total (N = 256)
Female (%)	166 (64.84)
Age (years)	35.00 (29.00 43.75)
Height (cm)	168.00 (162.25, 175.00)
Weight (kg)	107.00 (91.00, 123.75)
BMI (kg/m^2^)	38.37 ± 7.24
Waist (cm)	117.62 ± 16.06
Hip (cm)	121.89 ± 14.46
Waist-to-hip ratio	0.96 ± 0.08
T2DM (%)	148 (57.8)
Metformin use (%)	75 (29.30)
Hypertension (%)	119 (46.4)
SBP (mmHg)	138.08 ± 19.57
DBP (mmHg)	87.16 ± 15.28
Smoking history (%)	47 (18.36)
Alcohol consumption (%)	37 (14.45)
Type of surgery	
SG (%)	202 (78.91)
RYGB (%)	54 (21.09)

Abbreviations: BMI, body mass index; DBP, diastolic blood pressure; RYGB, Roux-en-Y gastric bypass; SBP, systolic blood pressure; SG, sleeve gastrectomy; T2DM, type 2 diabetes mellitus.

### 3.2 Postoperative weight loss

**Figure 1:**
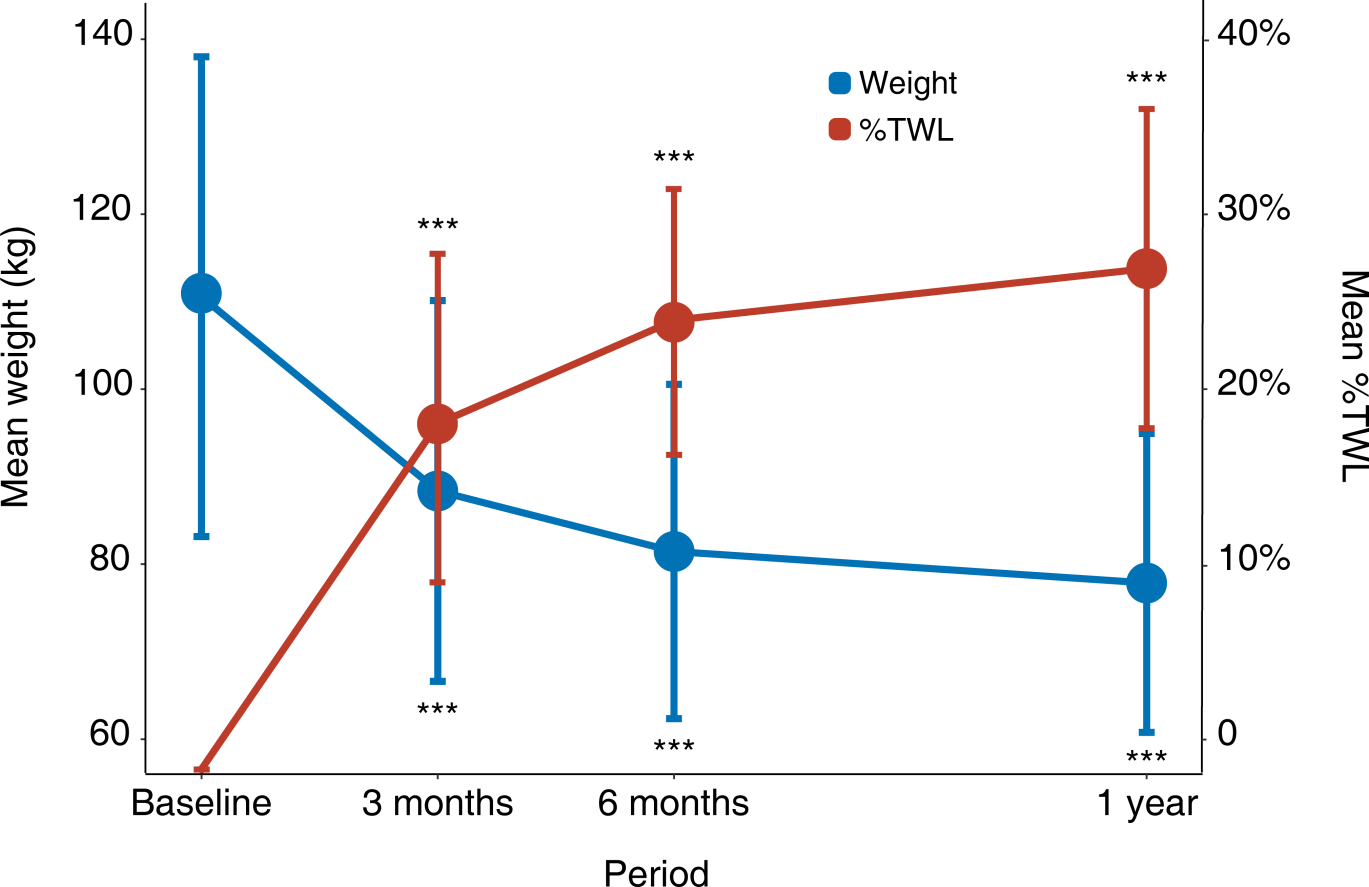
Weight loss after surgery. Error bars represent standard deviations. ^*^: *P* < 0.05, ^**^: *P* < 0.01, ^***^: *P* < 0.001 for each visit time compared with baseline. %TWL, percentage of total weight loss.

The body weight of all the patients decreased from 110.57 ± 27.42 kg at baseline to 88.37 ± 21.76 at 3 months, 81.45 ± 19.11 at 6 months, and 77.86 ± 17.08 kg at 1 year. The %TWL was 20.08 ± 6.39% at 3 months, 25.57 ± 7.59% at 6 months, and 28.62 ± 9.12% at 1 year, respectively (**Figure 1**).

### 3.3 Alterations in thyroid function and other biochemical markers

**Figure 2:**
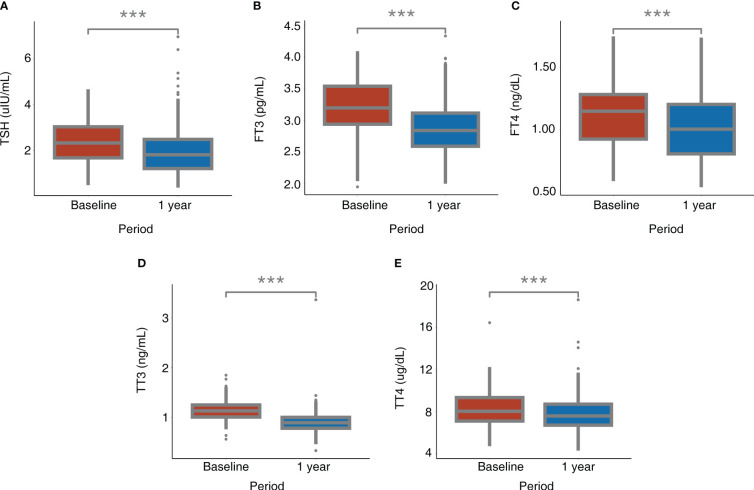
Alterations of TSH and thyroid hormones after bariatric surgery. **(A)** TSH; **(B)** FT3; **(C)** FT4; **(D)** TT3; **(E)** TT4. ^*^: *P* < 0.05, ^**^: *P* < 0.01, ^***^: *P* < 0.001 for 1 year after surgery compared with baseline. FT3, free triiodothyronine; FT4, free thyroxine; TSH, thyroid-stimulating hormone; TT3, total triiodothyronine; TT4, total thyroxine.

**Table 2** and **Figure 2** present the serum levels of TSH and thyroid hormones at baseline and 1 year after bariatric surgery. The serum level of TSH decreased from 2.33 (1.67, 3.04) uIU/mL to 1.82 (1.21, 2.50) uIU/mL (*P* < 0.001). The serum level of FT3 decreased from 3.23 ± 0.42 pg/mL to 2.89 ± 0.41 pg/mL (*P* < 0.001). The serum level of FT4 decreased from 1.11 ± 0.25 ng/dL to 1.02 ± 0.25 ng/dL (*P* < 0.001). The serum level of TT3 decreased from 1.13 (1.00, 1.25) ng/mL to 0.89 (0.78, 1.00) ng/mL (*P* < 0.001). The serum level of TT4 decreased from 8.28 ± 1.69 ug/dL to 7.82 ± 1.68 ug/dL (*P* < 0.001).

**Table 2:** Alterations in TSH, thyroid hormones, and other biochemical variables.

Variables	Baseline	1 year	*P*
TSH (uIU/mL)	2.33 (1.67, 3.04)	1.82 (1.21, 2.50)	<0.001
FT3 (pg/mL)	3.23 ± 0.42	2.89 ± 0.41	<0.001
FT4 (ng/dL)	1.11 ± 0.25	1.02 ± 0.25	<0.001
TT3 (ng/mL)	1.13 (1.00, 1.25)	0.89 (0.78, 1.00)	<0.001
TT4 (ug/dL)	8.28 ± 1.69	7.82 ± 1.68	<0.001
Other biochemical variables			
FPG (mmol/L)	6.26 (5.36, 9.15)	4.82 (4.38, 5.24)	<0.001
HbA1c (%)	6.30 (5.53, 8.38)	5.33 (5.10, 5.70)	<0.001
TC (mmol/L)	4.89 ± 1.00	4.48 ± 0.96	<0.001
TG (mmol/L)	1.79 (1.33, 2.35)	0.89 (0.69, 1.21)	<0.001
HDL-C (mmol/L)	1.03 (0.88, 1.20)	1.24 (1.08, 1.44)	<0.001
LDL-C (mmol/L)	3.06 ± 0.80	2.58 ± 0.76	<0.001

FPG, fasting plasma glucose; FT3, free triiodothyronine; FT4, free thyroxine; HbA1c, glycated hemoglobin; HDL-C, high-density lipoprotein cholesterol; LDL-C, low-density lipoprotein cholesterol; TC, total cholesterol; TG, total triglyceride; TSH, thyroid-stimulating hormone; TT3, total triiodothyronine; TT4, total thyroxine.

The decline was also observed in serum levels of FPG, HbA1c, TC, TG, LDL-C, and HDL-C with statistical significance (*P* < 0.001 for all, **Table 2**).

### 3.4 The association between ΔTSH and %TWL

**Figure 3:**
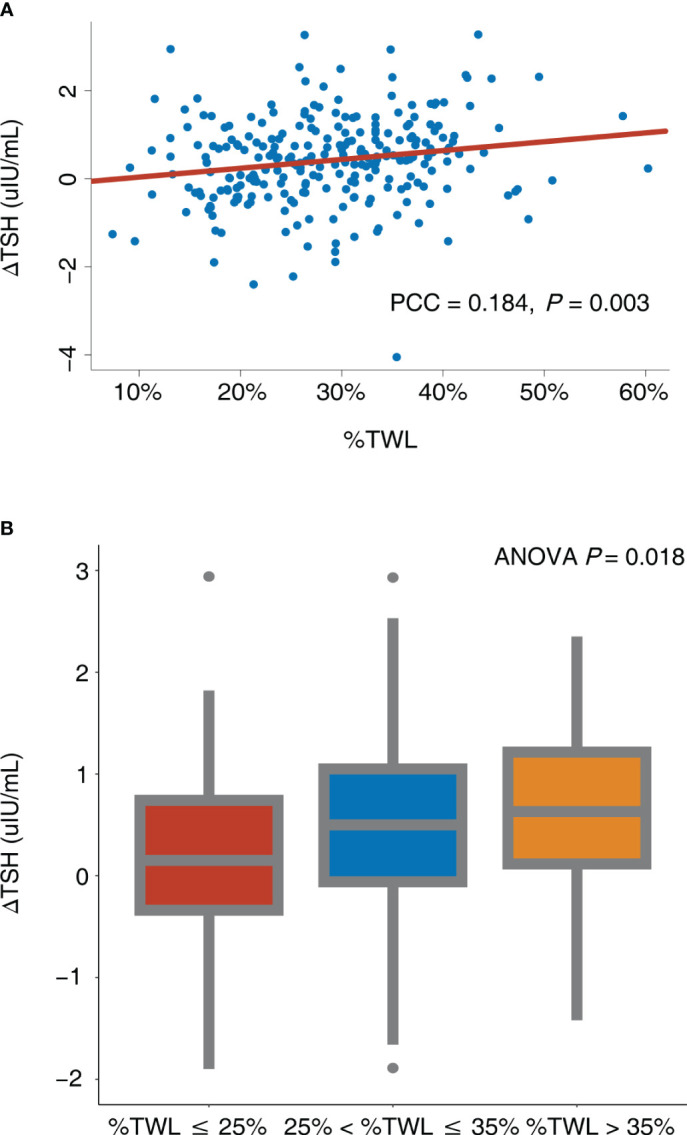
Correlation between ΔTSH and %TWL. **(A)** %TWL was used as continuous variable; **(B)** %TWL was used as categorical variable. ANOVA, analysis of variance; PCC, Pearson correlation coefficient; TSH, thyroid-stimulating hormone; %TWL, percentage of total weight loss.

According to the Pearson correlation analysis (**Figure 3A**), ΔTSH exhibited a significant positive correlation with %TWL (Pearson correlation coefficient = 0.184, *P* = 0.003), indicating that the more the weight loss, the more the TSH decrease. After further dividing the %TWL into 3 groups, ΔTSH levels differed significantly between groups (*P* = 0.018) and increased as the %TWL increased (**Figure 3B**).

**Table 3** presents the association between ΔTSH and %TWL/%TWL group. The %TWL revealed a significant linear relationship with the ΔTSH (β = 0.184 [95% confidence interval (CI), 0.063 – 0.306], *P* = 0.003) according to the univariable linear regression (Model 1). After accounting for age, sex, and BMI, the similar correlation was observed (β = 0.144 [95% CI, 0.017 – 0.271], *P* = 0.026) (Model 2). After making further adjustment for baseline sociodemographic variables, the %TWL still remained correlated with ΔTSH (β = 0.180 [95% CI, 0.048 – 0.312], *P* = 0.008) (Model 3).

**Table 3:** Standardized β and 95% CIs of ΔTSH according to %TWL.

	Model 1		Model 2		Model 3	
Variables	β (95% CI)	*P*	β (95% CI)	*P*	β (95% CI)	*P*
%TWL	0.184 (0.063 - 0.306)	0.003	0.144 (0.017 - 0.271)	0.026	0.180 (0.048 - 0.312)	0.008
%TWL group	0.176 (0.054 - 0.298)	0.005	0.136 (0.009 - 0.263)	0.035	0.153 (0.019 - 0.287)	0.025

Model 1: unadjusted. Model 2: adjusted for sex, age, and BMI. Model 3: adjusted for sex, age, BMI, waist-to-hip ratio, type of surgery, T2DM, metformin use, hypertension, smoking history, and alcohol consumption.

CI, confidence interval; TSH, thyroid-stimulating hormone; %TWL, percentage of total weight loss.

The %TWL group had a significant linear relationship with ΔTSH (β = 0.176 [95% CI, 0.054 – 0.298], *P* = 0.005) according to univariable linear regression (Model 1). After adjusting for age, sex, and BMI, the %TWL group still presented a significant relationship with ΔTSH (β = 0.136 [95% CI, 0.009 – 0.263], *P* = 0.035) (Model 2). In the fully adjusted model, the %TWL group was also found to be an independent predictor for ΔTSH (β = 0.153 [95% CI, 0.019 – 0.287], *P* = 0.025) (Model 3).

**Figure 4:**
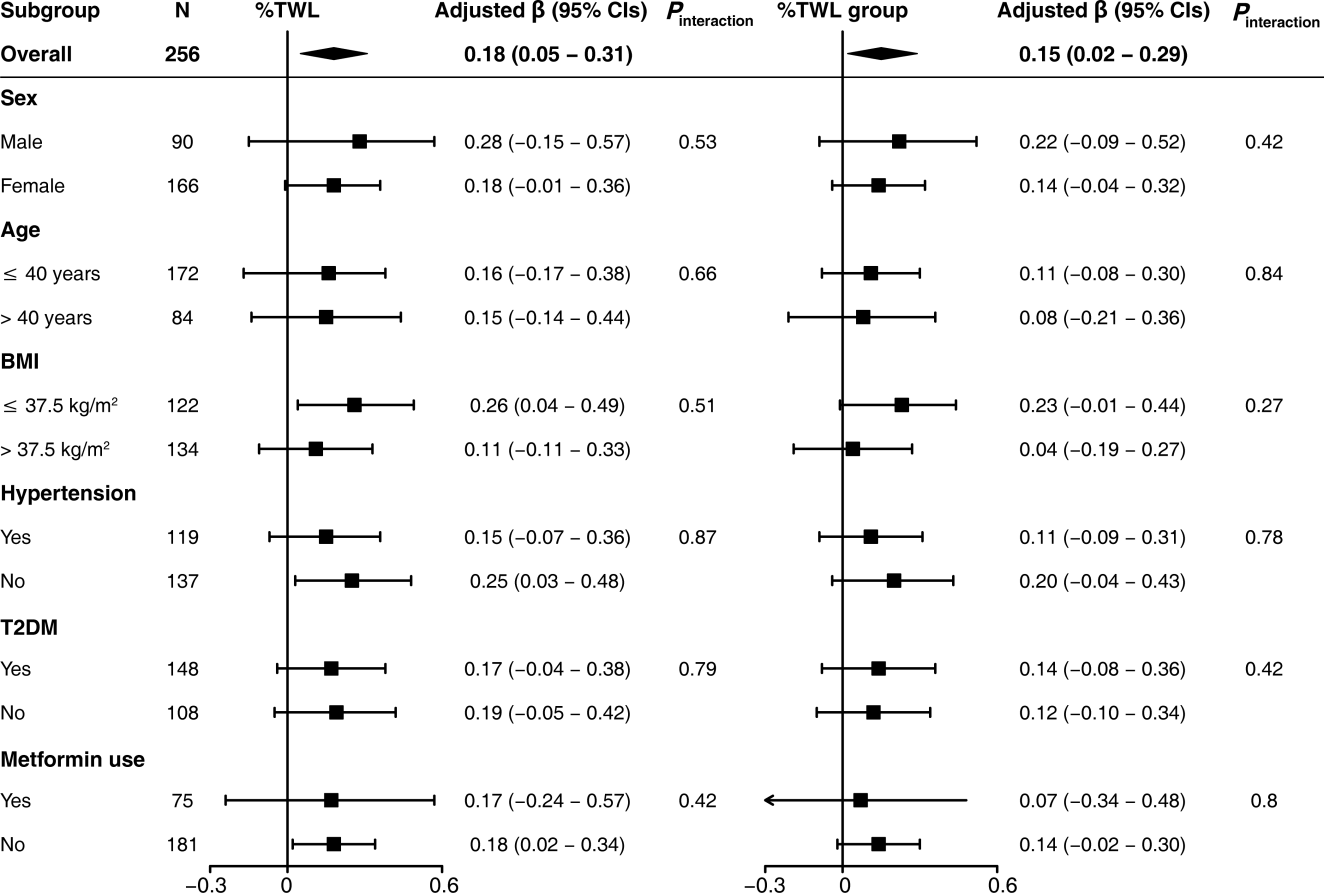
Subgroup analysis for the association between ΔTSH and %TWL. The %TWL was used as continuous variable (%TWL) and categorical variable (%TWL group). Each subgroup analysis adjusted for sex, age, BMI, waist-to-hip ratio, type of surgery, hypertension, T2DM, metformin use, smoking history, and alcohol consumption. BMI, body mass index; T2DM, type 2 diabetes mellitus; %TWL, percentage of total weight loss.

In addition, patients in subgroup analyses were categorized based on sex (male or female), age (≤ 40 years or >40 years), BMI (≤ 37.5 kg/m^2^ or >37.5 kg/m^2^), T2DM (yes or no), metformin use (yes or no), and hypertension (yes or no). No significant interactions between %TWL and these potential covariates were observed (all *P* values for interaction > 0.05) (**Figure 4**).

## 4 Discussion

Thyroid hormones play an essential role in regulating dietary intake and energy expenditure and therefore interact with body weight (26). Patients with obesity frequently suffer from thyroid dysfunction, which has the potential to exacerbate the obese state. However, the effect of bariatric surgery, a powerful method of weight loss, on thyroid function is not known with certainty. The main results of this study showed that TSH, FT3, FT4, TT3, and TT4 decreased significantly 1 year after bariatric surgery in euthyroid patients. TSH reduction was independently correlated with %TWL, with greater weight loss correlating to greater TSH reduction.

Individuals with obesity have been found to have elevated TSH when compared to normal-weight controls, as well as thyroid hormones (27–30). According to a bidirectional Mendelian randomization study by Wang et al. (31), it is more likely that obesity causes elevated TSH than that elevated TSH causes weight gain. Obesity was found to reduce the expression of TSH receptors on adipocytes, which may induce the downregulation of thyroid hormone receptors and action and further increase TSH (32). Moreover, the elevation of TSH can be mediated by compensatory activation of the hypothalamus-pituitary-thyroid (HPT) axis in response to an increase in leptin in adipose tissue (33, 34). Thus, the combined effect of peripheral thyroid hormone resistance and central leptin regulation increases TSH in plasma and has detrimental impacts on organs beyond the thyroid.

Changes in TSH and thyroid hormones after bariatric surgery remain controversial. We analyzed the data of 256 Chinese patients and revealed a significant decrease in TSH and other thyroid hormones (FT3, FT4, TT3, and TT4) 1 year after SG and RYGB. The majority of studies on patients with normal thyroid function or subclinical hypothyroidism have yielded similar outcomes (13, 18). In contrast, Dall’Asta et al. (17) examined the effect of gastric banding on thyroid hormones and found no postoperative change in TSH. Zhang et al. (35) and MacCuish et al. (16) investigated the variations in thyroid function following RYGB and discovered that TSH remained steady. FT3 and FT4 have also been reported to be elevated, unchanged, or decreased after bariatric surgery in previous studies, with the varying results primarily attributable to differences in ethnic groups, surgical procedures, and sample sizes (9, 14, 19, 36, 37). However, the present study included, to the best of our knowledge, the largest number of Chinese euthyroid patients with obesity and comprehensively analyzed the two most common bariatric surgical procedures (SG and RYGB), so it may be able to provide more reliable data for Chinese patients.

Although a few studies failed to identify a relationship between TSH decline and weight loss after bariatric surgery (19, 37–40), most studies initially explored a positive correlation using correlation analysis (14, 17, 19, 41). Neves et al. (42) analyzed preoperative and 1-year postoperative thyroid function in 949 euthyroid patients who underwent SG, RYGB, or gastric banding and revealed that a decrease in TSH was significantly associated with excessive body weight loss. Juiz-Valiña et al. (27) conducted a study comparing the TSH levels of 129 euthyroid patients before and after SG and RYGB and observed that TSH reduction was associated with excessive BMI loss. Moreover, a recent prospective study by Kamal et al. (43), involving 106 euthyroid patients undergoing SG has indicated a positive linear relationship between %TWL and ΔTSH. Using Pearson correlation analysis, we also discovered a significant correlation between ΔTSH and %TWL (PCC = 0.184, *P* = 0.003), which is consistent with previous findings. Similar results were observed after dividing %TWL into 3 groups, with greater TSH variations as %TWL increased. To confirm the relationship between ΔTSH and %TWL, we performed multivariable stepwise linear regression. After adjusting for several covariates, both %TWL and %TWL groups were found to be independent predictors of TSH decline, suggesting that changes in thyroid function after bariatric surgery are mediated by surgically induced weight loss, with more weight loss resulting in better thyroid function improvement.

How weight loss after bariatric surgery affects TSH and thyroid hormones is not yet fully understood. One viewpoint proposed that the improvement of thyroid function is mediated by weight loss independently, rather than being exclusively attributed to the effects of bariatric surgery (44). This was supported by the fact that weight loss induced by lifestyle changes can also reduce TSH levels (45). Although there are no studies directly comparing the effects of bariatric surgery and lifestyle interventions on thyroid function, we concluded by comparing differing degrees of weight loss after surgery that %TWL > 35% was associated with greater TSH changes than 25 ≤ %TWL < 35% and greater than %TWL < 25%. Given that bariatric surgery is the most effective method of weight loss currently available, its effect on thyroid function should be the most significant.

Various potential factors may cause the alteration in thyroid function after bariatric surgery. First, the tremendous decrease in fat mass and leptin released from adipocytes after bariatric surgery not only reduces the stimulation of the HPT axis but also inhibits the peripheral conversion of T4 to T3, resulting in a decrease in TSH and thyroid hormones, which may be the main mechanism for the alteration of thyroid function after bariatric surgery (46–48). Second, decreased expression of the TSH receptor due to obesity can be reversed by weight loss, thereby reducing peripheral thyroid hormone resistance (32). Third, the growth hormone is considered as an important factor that could regulate the HPT axis and decreased significantly in patients with obesity. After bariatric surgery, the growth hormone rises and lowers TSH through the HPT axis (44, 49). Fourth, the body inflammation state declined after bariatric surgery, which has been reported to be associated with the reduction of TSH, resulting in the improvement of thyroid hormone resistance after surgery (50). Fifth, bariatric surgery can inhibit the activity of B and T lymphocytes and reduce the circulating levels of thyroid autoimmune antibodies and various inflammatory cytokines, which may protect the thyroid gland from inflammatory injuries and the forced release of stored thyroid hormones (51). Finally, improvements in glycemic and lipid profiles after bariatric surgery have also been reported to play a role in promoting a decrease in TSH (19, 41, 52), which aligns with our findings as the FPG, HbA1c, TC, TG, LDL-C, and HDL-C all declined significantly after surgery.

From a clinical perspective, the present study identified a positive correlation between bariatric surgery-induced weight loss and TSH reduction in patients with normal thyroid function, providing physiological encouragement for the clinical therapeutic strategy of surgical treatment for obesity-related thyroid dysfunction. TSH will decrease following weight loss after bariatric surgery in patients with TSH levels approaching the upper normal limit, without the need for additional thyroid hormone-lowering medication. For patients with hypothyroidism, the current study was in accordance with the previous study that TSH would decline after bariatric surgery, supporting the view that the thyroid replacement medication dosage might be reduced (53). If enhanced efficacy is needed, weight management, such as dietary control and exercise interventions, can be strengthened postoperatively to increase %TWL, which leads to a further decrease in TSH.

Some limitations to our study should be noted. Firstly, since this was a retrospective, single-center study, there was a selection bias. Secondly, the study population comprised only Chinese patients with obesity, so it is unknown whether these results can be generalized to Western populations. Thirdly, antithyroid antibodies and cytokines such as leptin were not routinely measured in the present study, so we may not be able to further explore the specific mechanism underlying the impact of weight loss on TSH levels. Fourthly, due to a significant portion of patients lacking thyroid function-related data at 3 months and 6 months after surgery, we have not included that for analysis. Finally, the follow-up period of this study was only 1 year; hence, the alterations in thyroid function after 1 year and the influence of weight regain on thyroid function cannot be studied. Future research should establish multicenter, prospective, and long-term follow-up cohorts to obtain more reliable data.

In conclusion, our study revealed a significant reduction in TSH, FT3, FT4, TT3, and TT4 in Chinese euthyroid patients with obesity 1 year after bariatric surgery. Weight loss following SG and RYGB will improve thyroid function. The more weight loss occurs, the more TSH decreases.

## Data availability statement

The original contributions presented in the study are included in the article/**Supplementary Materials**, further inquiries can be directed to the corresponding author/s.

## Ethics statement

The studies involving humans were approved by Institutional Review Board of China-Japan Friendship Hospital. The studies were conducted in accordance with the local legislation and institutional requirements. The human samples used in this study were acquired from primarily isolated as part of your previous study for which ethical approval was obtained. Written informed consent for participation was not required from the participants or the participants’ legal guardians/next of kin in accordance with the national legislation and institutional requirements.

## Author contributions

ZT: Data curation, Formal analysis, Software, Visualization, Writing – original draft, Writing – review & editing. YN: Conceptualization, Methodology, Writing – review & editing, Funding acquisition, Writing – original draft. ZL: Writing – review & editing, Data curation, Funding acquisition. PW: Data curation, Investigation, Writing – review & editing. NZ: Data curation, Writing – review & editing. XH: Data curation, Writing – review & editing. AP: Software, Writing – review & editing. BL: Methodology, Writing – review & editing. HM: Conceptualization, Supervision, Writing – review & editing, Funding acquisition.
