# Supplementary material for: Total weight loss induces the alteration in thyroid function after bariatric surgery
Source: Front Endocrinol (Lausanne). 2024 Jan 30;15:1333033. doi: 10.3389/fendo.2024.1333033 (PMC10861714; doi:10.3389/fendo.2024.1333033)
Supplement: Supplementary file 1 [file Table_1.docx]

| Supplementary Table 1. Normal ranges for biochemical markers. | |
| --- | --- |
| Markers | Normal range |
| TSH (uIU/mL) | 0.50 - 4.80 |
| FT3 (pg/mL) | 1.95 - 4.26 |
| FT4 (ng/dL) | 0.58 - 1.75 |
| TT3 (ng/mL) | 0.56 - 1.85 |
| TT4 (ug/dL) | 4.60 - 16.50 |
| FPG (mmol/L) | 3.61 - 6.11 |
| HbA1c (%) | 4.00 – 6.00 |
| TC (mmol/L) | < 5.20 |
| TG (mmol/L) | < 1.70 |
| HDL-C (mmol/L) | 1.00 - 1.20 |
| LDL-C (mmol/L) | ≤ 3.40 |
| Abbreviations: FPG, fasting plasma glucose; FT3, free triiodothyronine; FT4, free thyroxine; HbA1c, glycated hemoglobin; HDL-C, high-density lipoprotein cholesterol; LDL-C, low-density lipoprotein cholesterol; TC, total cholesterol; TG, total triglyceride; TSH, thyroid-stimulating hormone; TT3, total triiodothyronine; TT4, total thyroxine. | |
